# Poor family relationships in adolescence as a risk factor of in-patient psychiatric care across the life course: A prospective cohort study

**DOI:** 10.1177/1403494820902914

**Published:** 2020-02-03

**Authors:** Susanne Alm, Sara Brolin Låftman, Fredrik Sivertsson, Hannes Bohman

**Affiliations:** 1 Swedish Institute of Social Research, Stockholm University, Sweden; 2 Department of Public Health Sciences, Centre for Health Equity Studies (CHESS), Stockholm University, Sweden; 3 Department of Criminology, Stockholm University, Sweden; 4 Department of Neuroscience, Child and Adolescent Psychiatry, Uppsala University, Sweden; 5 Department of Women’s and Children’s Health, Akademiska University Hospital, Sweden; 6 Stockholm Health Care Services, Stockholm County Council, Sweden

**Keywords:** Family conflict, family discord, mental illness, cohort, longitudinal, prospective

## Abstract

*Background:* Previous research has shown that poor family relations in childhood are associated with adverse mental health in adulthood. Yet, few studies have followed the offspring until late adulthood, and very few have had access to register-based data on hospitalisation due to psychiatric illness. The aim of this study was to examine the association between poor family relations in adolescence and the likelihood of in-patient psychiatric care across the life course up until age 55. *Methods:* Data were derived from the Stockholm Birth Cohort study, with information on 2638 individuals born in 1953. Information on family relations was based on interviews with the participants’ mothers in 1968. Information on in-patient psychiatric treatment was derived from administrative registers from 1969 to 2008. Binary logistic regression was used. *Results:* Poor family relations in adolescence were associated with an increased risk of later in-patient treatment for a psychiatric diagnosis, even when adjusting for other adverse conditions in childhood. Further analyses showed that poor family relations in adolescence were a statistically significant predictor of in-patient psychiatric care up until age 36–45, but that the strength of the association attenuated over time. ***Conclusions:* Poor family relationships during upbringing can have serious negative mental-health consequences that persist into mid-adulthood. However, the effect of poor family relations seems to abate with age. The findings point to the importance of effective interventions in families experiencing poor relationships.**

## Introduction

Social relationships in the family of origin are important for children’s health and development, and dysfunctional family relationships may have long-lasting adverse consequences. Previous research has shown that adverse family relations during upbringing are associated with an increased risk of poor mental health up until mid-adulthood. A systematic review of prospective studies reported that adverse family relationships in childhood were associated with an increased risk of psychiatric disorders in later life [1]. In particular, this concerned parental physical and sexual abuse and neglect. More common and less severe types of dysfunctional family relationships, such as poor emotional responsiveness and family discord, were also predictive of later psychiatric disorders, but in order to draw more robust conclusions about causality in such associations, further research was called for [1]. Indeed, more recent studies have demonstrated poor family relationships during upbringing to be linked with an increased risk of mental-health problems later in life [2–4] and even with premature death [5]. However, studies following individuals as long as until late adulthood are scarce. In addition, few earlier studies have used register-based data on hospitalisation due to psychiatric illness. One important advantage with register data is that it lacks problems with non-response, which may be substantial in studies using self-reported measures of mental ill health. Further, register data for in-patient care may be expected to capture severe forms of mental ill health, which would be of high importance to prevent. The aim of the current study was to examine the association between poor family relations in adolescence and in-patient psychiatric care up until age 55, even when taking other adverse conditions in childhood into account.

## Methods

### Data

The data came from the Stockholm Birth Cohort study (SBC). The SBC database was set up by combining two de-identified data sets [6,7]. The first is the Metropolitan Study database, covering all individuals born in 1953 and living in Stockholm 10 years later (*n*=15,117) [8]. The Metropolitan Study database includes both survey and register data. When the members of the cohort were 13 years old, they were asked to complete a questionnaire in school, with questions on, for example, friendships, interest in school and aspirations. In 1968, the Family Study was carried out. This study included interviews with mothers of a stratified subsample of original cohort members who were still residing in metropolitan Stockholm as of 1 November 1967. The questions to the mothers concerned, for example, their attitudes to education but also their estimations of the relations within the family. Out of 4021 sampled individuals for the Family Study, interviews were completed with 91% (*n*=3651). Register data within the Metropolitan Study include information on, for example, social problems in the family of origin from the social authorities, but also, for instance, income.

The second data set, to which the data from the Metropolitan Study have been linked, is the so-called Work and Mortality Database (WMD), which comprises register data on all individuals living in Sweden in 1980 and/or 1990, and who were born before 1985. The WMD includes information on, inter alia, income, receipt of social welfare, in-patient care and mortality, and data are available up to 2008 [6]. Matching without identification was possible, since the two databases contained a number of identical variables. The variables were used to form unique series for each observation in both data sets, after which matching on the series was made. With the matched data material, we were able to follow the individuals born in 1953 until they were 55 years old.

More information about the SBC study, including the Family Study and the WMD, can be found at https://www.stockholmbirthcohort.su.se.

This study uses the information from the subsample of mothers interviewed in the Family Study within the Metropolitan Study, together with register information from the WMD (*n*=3476). The study sample is further restricted to those respondents who at the age of 15 were living with both their biological parents and who had at least one sibling (*n*=2638).

The Reproduction of Inequality through Linked Lives (RELINK) project, of which the study is a part, has been ethically approved by the Regional Ethical Review Board of Stockholm (2017/34-31/5 and 2017/684-32).

### Measures

#### Dependent variable

In-patient psychiatric care: The measures were based on data from the Hospital Discharge Register on individuals admitted for in-patient treatment for a psychiatric diagnosis at 16–55 years of age. The following codes were used: ICD-8 (1969–1986): Chapter V, codes 290–309; IC-9 (1987–1996): Chapter V, codes 290–314; ICD-10 (1997–2008): Chapter V, codes F00–F69.

#### Independent variable

Family relations: The measure was constructed from four questions asked to each participant’s mother in the Family Study in 1968: (a) ‘How would you describe the relationship between you and your son/daughter?’; (b) ‘How would you describe the relationship between your husband and your son/daughter?’; (c) ‘How would you describe the relationship between your son/daughter and his/her siblings?’; and (d) ‘How would you describe the relationship between you and your husband?’. For all questions, there were five response categories: (a) ‘very good’, (b) ‘rather good’, (c) ‘neither good nor bad’, (d) ‘rather bad’ and (e) ‘very bad’. The scores on the four questions were summed to an index ranging from 5 to 20, with higher values indicating better family relations. The index had good internal consistency (Cronbach’s α=0.75). Due to the skewed distribution, with a majority of respondents reporting ‘very good’ or ‘rather good’ familial relations, the index was divided into three categories to indicate good (scores 19–20), intermediate (scores 16–18) and poor (scores <16) family relations. Our main focus was on poor family relationships, and with this categorisation, approximately the decile with the lowest scores on the index were classified as having ‘poor’ family relations. The cut-off for poor family relations implied that this category had not necessarily reported ‘bad’ relations in an absolute sense. In a relative sense, however, their family relationships could be classified as poor. The remaining cases were divided into two groups of about equal size to capture ‘good’ and ‘intermediate’ family relationships, again in a relative sense. This trichotomised measure of family relationships has been used previously [5].

#### Controls

Sex: 0=male, 1=female.

Household social class: The indicator was based on the father’s or, if information on this was missing, on the mother’s occupation. The variable was coded into three categories: (a) upper non-manual; (b) lower non-manual, self-employed and farmers; and (c) manual and unclassified. This information was collected for the Population Register in 1963 when the study participants were 10 years old.

Household economic poverty: The measure was based on register information from the Social Register and indicated receipt of social welfare in the family of origin in the years 1953–1965 when the study participants were 0–12 years old. The variable distinguishes those who did not receive any social welfare during the period (0) from those who received social welfare on at least one occasion during the period (1).

Contact with child services: If the family of origin had been in contact with child services as a consequence of the behaviour of the study participant at 7–12 years of age, the variable was coded 1, otherwise it was coded 0. Information was based on the Social Register.

Parental alcohol abuse: If (at least one of) the study participant’s parents had been registered by the social authorities for alcohol abuse at any time point during the period 1953–1965, the variable was coded 1, otherwise it was coded 0. Information was based on the Social Register.

Parental mental illness: If (at least one of) the study participant’s parents had been registered by the social authorities for mental illness at any time point during the period 1953–1965, the variable was coded 1, otherwise it was coded 0. Information was based on the Social Register.

### Statistical method

The number of individuals with experiences of psychiatric in-patient treatment was rather small: 7.6% of the study individuals during the entire study period when the individuals were aged 16–55 years. In addition, as few as 4.8% had experienced more than one event of in-patient psychiatric care. Due to these quite small numbers, binary logistic regression, distinguishing those with at least one event of inpatient care (coded 1) from those with no such experience (coded 0), was applied.

In a first step, predictors for in-patient psychiatric care during the full age period of 16–55 years of age were analysed. Subsequently, in order to investigate whether the association between poor family relationships and in-patient psychiatric care varied at different ages, a series of binary logistic regressions predicting in-patient psychiatric care at four different time periods – 16–25, 26–35, 36–45 and 46–55 years – were performed.

## Results

### Main results

Descriptive statistics of the study variables are presented in Table I. The proportion of individuals in the study sample who had experienced at least one event of in-patient psychiatric treatment each 10-year period was around 3%. As mentioned above, the corresponding share during the entire observation period, in those aged 16–55, was 7.6%. As regards family relations in adolescence, the mothers in the Family Study rated these rather positively, but according to our categorisation, 9.1% of the study participants were exposed to poor family relations. Finally, 12.3% suffered from economic poverty, whereas 1.7–2.9% scored positive on contact with child services, parental alcohol abuse and parental mental illness.

**Table I.** Descriptions of the study variables (*n*=2500–2638).

	*n*	%
Family relationships (index), *M, SD*	17.9	1.9
At least one event of in-patient psychiatric care		
16–25 years	74	2.8
26–35 years	77	3.0
36–45 years	97	3.8
46–55 years	87	3.5
At any time point (16–55 years)	190	7.6
Family relations		
Good	1270	48.1
Intermediate	1128	42.8
Poor	240	9.1
Sex		
Males	1355	51.4
Females	1283	48.6
Household social class		
Upper class/upper middle class	592	22.4
Intermediate/lower middle class/entrepreneur/farmer	1060	40.2
Working class/unclassified	986	37.4
Household economic poverty	325	12.3
Contact with child services	54	2.0
Parental alcohol abuse	45	1.7
Parental mental illness	76	2.9

In a next step, binary logistic regressions were performed, with results displayed in Table II. The first columns of the table display the unadjusted associations, while the columns to the right give the adjusted ones. Concerning the unadjusted associations, compared to those with good family relations, participants with intermediate and poor family relations were significantly more likely to have been taken in for psychiatric care (odds ratio (OR)=1.61, 95% confidence interval (CI) 1.17–2.23 and OR=2.59, 95% CI 1.63–4.11). As regards the control variables, all included factors with the exception of sex were significantly associated with the likelihood of having been in psychiatric in-patient care in the unadjusted analyses. Even more importantly, in the adjusted model, the association between family relations in adolescence and in-patient psychiatric care in adulthood remained strong and statistically significant (OR=1.54, 95% CI 1.11–2.14 for intermediate relations and OR=2.34, 95% CI 1.39–3.59 for poor relations). The same was true concerning household economic poverty, while the remaining associations were attenuated and turned non-significant in the full model.

**Table II.** Associations between family relations in adolescence and in-patient psychiatric care at ages 16–55 years.

	% in psychiatric care (*n*)	Unadjusted		Adjusted	
		OR	95% CI	OR	95% CI
Family relations					
Good (ref.)	5.6 (68)	1.00	–	1.00	–
Intermediate	8.7 (93)	**1.61**	1.17–2.23	**1.54**	1.11–2.14
Poor	13.3 (29)	**2.59**	1.63–4.11	**2.34**	1.39–3.59
Sex					
Males (ref.)	8.0 (101)	1.00	–	1.00	–
Females	7.2 (89)	0.89	0.66–1.20	0.86	0.63–1.16
Household social class					
Upper non-manual (ref.)	5.6 (32)	1.00	–	1.00	–
Interm./lower non manual/entrepreneur/farmer	6.5 (66)	1.17	0.76–1.81	1.03	0.66–1.59
Manual worker	10.0 (92)	**1.87**	1.23–2.83	1.28	0.82–2.00
Household economic poverty	17.2 (50)	**3.07**	2.16–4.35	**2.49**	1.64–3.78
Contact with child services	17.1 (7)	**2.56**	1.12–5.86	1.48	0.62–3.55
Parental alcohol abuse	17.6 (6)	**2.66**	1.09–6.50	1.18	0.46–3.06
Parental mental illness	17.6 (12)	**2.71**	1.43–5.16	1.09	0.53–2.22

Odds ratios (OR) and 95% confidence intervals (95% CI) from binary logistic regressions. Results statistically significant at the 5% level are reported in bold. *n*=2500.

To investigate whether the association between poor family relationships and in-patient psychiatric care varied at different ages, a series of binary logistic regressions predicting in-patient psychiatric care at four different time periods was performed (Table III). Each of the regressions excluded individuals who were deceased at the end of each 10-year period. The adjusted analyses show that poor family relations were associated with an increased likelihood of in-patient psychiatric care at 16–25 years (OR=4.54, 95% CI 2.34–8.83), 26–35 years (OR=3.14, 95% CI 1.62–6.10) and 36–45 years (OR=1.94, 95% CI 1.02–3.70), even when controlling for other adverse childhood experiences. The association between poor family relations in adolescence and in-patient psychiatric care at 46–55 years of age was, however, not statistically significant (OR=1.70, 95% CI 0.84–3.47).

**Table III.** Associations between family relations in adolescence and in-patient psychiatric care at ages 16–25, 26–35, 36–45 and 46–55 years.

	% in psychiatric care (*n*)	Unadjusted		Adjusted	
		OR	95% CI	OR	95% CI
*16–25 years (*n*=2614)*					
Family relations					
Good (ref.)	1.6 (20)	1.00	–	1.00	–
Intermediate	3.1 (35)	**2.01**	1.15–3.49	**1.90**	1.09–3.32
Poor	8.0 (19)	**5.40**	2.84–10.29	**4.54**	2.34–8.83
*26–35 years (*n*=2592)*					
Family relations					
Good (ref.)	2.0 (25)	1.00	–	1.00	–
Intermediate	3.2 (35)	1.61	0.96–2.70	1.55	0.92–2.63
Poor	7.2 (17)	**3.83**	2.03–7.21	**3.14**	1.62–6.10
*36–45 years (*n*=2556)*					
Family relations					
Good (ref.)	2.8 (35)	1.00	–	1.00	–
Intermediate	4.3 (47)	1.55	1.00–2.43	1.46	0.93–2.29
Poor	6.6 (15)	**2.42**	1.30–4.52	**1.94**	1.02–3.70
*46–55 years (*n*=2500)*					
Family relations					
Good (ref.)	2.8 (34)	1.00	–	1.00	–
Intermediate	3.9 (42)	1.43	0.90–2.26	1.39	0.88–2.22
Poor	5.0 (11)	1.85	0.92–3.70	1.70	0.84–3.47

ORs and 95% CI from binary logistic regressions. Results statistically significant at the 5% level are reported in bold.

### Sensitivity analyses

Subsequently, a series of sensitivity analyses was performed to test the robustness of the results. First, as was demonstrated in the age-specific binary logistic regression analyses presented in Table III, the association between poor family relationships and in-patient psychiatric treatment attenuated over time. Theoretically, this could be the result of a tendency for those with the poorest family relations in adolescence to die earlier than others and thus to leave the cohort. To test this, all age-specific logistic regression models were rerun, restricting the sample to only those still alive at age 55 (see Table AI in the Supplemental material). The pattern in these analyses was very similar to the original ones (presented in Table III). In other words, the analysis strengthens the interpretation that the detrimental effect of poor family relations in adolescence attenuates over the lifespan, at least concerning its influence on the risk of in-patient psychiatric treatment.

In a second set of sensitivity analyses, the main independent variable, that is, the summary index of family relations, was broken down into its four original components: relationship between (a) mother and child, (b) father and child, (c) siblings and (d) mother and father. In these analyses, for each type of relationship, the categories ‘neither good nor bad’, ‘rather bad’ and ‘very bad’ were coded as a ‘poor’ relation; ‘rather good’ was coded as ‘intermediate’; and ‘very good’ (which served as the reference category) was coded as a ‘good’ relation (see Table AII in the Supplemental material). For all types of relations, the coefficients pointed in the expected direction, albeit to differing degrees. For siblings, the effect did not reach significance, and concerning the mother–child relation, the effect of poor family relations was unclear due to a large standard error. The strongest and most reliable effect was found for the father–child relationship. Since the coefficients for all types of relationships were in the expected direction, and since the index showed good reliability, the measure was kept in its original format.

In a third set of sensitivity analyses, we sought not only to assess the robustness of the results from the binary logistic regressions, but also to scrutinise whether poor family relationships were associated with a greater number of in-patient psychiatric care events. To this end, we applied negative binomial (NB) regression modelling to the data on the whole study period (see Table AIII in the Supplemental material). The *nbreg* command in Stata 15 was used. NB regression is a commonly used method for overdispersed count data, since it allows for independent specification of the mean and the variance. As a result, it produces larger standard errors than, for example, Poisson regression. The regression coefficients were exponentiated into incidence rate ratios (IRR), which are to be interpreted as the expected change in the rate of inpatient-care events with one step increase in the independent variable (or for a given group relative to the reference group when interpreting the dummy variables). The very large standard errors estimated for some of the control variables resulted in some non-significant associations, but concerning the main independent factor (i.e. family relations), the results were similar to those produced by the binary logistic regression models but additionally showed that intermediate or poor family relations in adolescence (compared to good family relations) were associated with a greater number of treatment events. Expressed in IRRs, the rate of inpatient psychiatric care for those with intermediate family relations was 2.58 times higher, while for those with poor family relations it was 3.30 times higher, compared to those with good family relations.

In a final set of sensitivity analyses, the psychiatric diagnoses were divided into four categories to investigate whether the associations were similar across different disorders. We distinguished between (a) substance abuse diagnoses, (b) mood and anxiety disorders, (c) psychoses and related disorders (e.g. schizophrenia) and (d) others. The results (see Table AIV in the Supplemental material) showed that the estimates were in the expected direction for all types of disorders but were particularly strong (and statistically significant) for substance abuse diagnoses and psychoses and related disorders.

## Discussion

The current study showed that poor family relations in adolescence were associated with an increased likelihood of in-patient treatment for a psychiatric diagnosis in adulthood, even when adjusting for other adverse conditions in childhood. The study reflects findings from prior research on poor family relationships and adverse mental health [1–4], but also contributes with new knowledge by focusing on hospitalisation, capturing severe mental-health problems and following individuals up until late adulthood.

The association between poor family relationships in childhood and psychiatric in-patient care in adulthood may be understood through different mechanisms. First, poor family relationships imply an increased risk of stress, which may lead to an increased susceptibility of health problems [9]. Another potential mechanism is related to the fact that poor family relationships in childhood tend to be associated with economic adversity in adulthood via lower education [10]. Indeed, the inverse association between socio-economic disadvantage and mental health is well established [11]. Further, poor family relationships in childhood may also lead to dysfunctional social relationships in adulthood [12,13]. The lack of supportive social relations is, in turn, associated with an increased risk of various types of health problems, including depressive symptoms [14].

Due to the long follow-up period with the opportunity to track individuals up until 55 years of age, we were able to assess the association between poor family relationships and in-patient psychiatric care at different ages. The results showed that the association remained robust up until the age of 36–45 years. However, for every 10-year period, the strength of the association was attenuated, indicating that the potential effect of poor family relations in adolescence on psychiatric disorder in later life may wear off over time. That the association seemed to wane off during the life course instead of worsening is a gratifying finding. This finding was somewhat unexpected and has, to the best of our knowledge, not been shown previously. An interpretation is that adverse mental health as a result of unfavourable interpersonal experiences in adolescence can heal with extended time. This could be the result of the effectiveness of in-patient mental-health care or other health care consumed. Another possibility could be an inherited ability in the individual to heal mentally with age. However, although the present study showed that the association between poor family relationships in childhood and in-patient psychiatric care was weaker as the individuals grew older, it is possible that adverse family relationships in childhood may have other harmful consequences that last into late adulthood. Indeed, prior studies have reported links between poor parent–child relationships in childhood and physical health problems in mid-adulthood [3,9], and a recent study based on data from the SBC showed poor family relationships in adolescence to be linked with an increased risk of premature death up until late adulthood [5].

This study benefited from prospective data material derived from a cohort of individuals born in 1953, with survey information on family relationships collected in the 1960s and official register information on in-patient psychiatric care linked up until 2008. The fact that our data include official register information on in-patient psychiatric care is a strength, since it is an indicator of severe mental ill health that is not easily captured by self-reports. An additional strength is that there is no attrition. Nevertheless, using this measure also means that we capture only the severe end of mental ill health, and hence we cannot draw the conclusion that poor family relationships are also associated with milder forms of mental ill health. The rich information on adverse childhood conditions enabled us to adjust for important confounders, strengthening the argument for an independent association between poor family relationships during upbringing and in-patient psychiatric care in later life. There are, however, also limitations. The study sample was restricted to participants living with a mother and a father and at least one sibling, thus limiting the possibility of generalisations to other family structures. That family relationships were rated by the participants’ mothers is another limitation, and information on family relationships collected from several family members would have been valuable. The phrasing of the questions on family relations was also rather broad in that interviewees were asked to evaluate the overall quality of the different relationships within the family. Furthermore, it should be emphasised that our measure of in-patient psychiatric care is likely to capture only a portion of those who suffered from mental disorders in that far from all individuals who need psychiatric care seek help [15]. It is, however, not evident whether, and if so how, this bias may have affected our findings. In order to gain more knowledge about the potential effects of poor family relationships in childhood on adverse mental-health outcome across life, studies of different types of mental-health measures are needed.

Finally, while the present study did not focus on interventions aiming to improve family relationships, such programmes can be beneficial [16] and may therefore possibly also hinder the long-term effect of poor family relationships on psychiatric disorder in the offspring.

## Conclusions

Poor family relationships during upbringing can have serious negative mental-health consequences that persist into mid-adulthood. The findings point to the importance of effective interventions in families that experience poor relationships. However, the finding that the association between poor family relations and severe mental disorders seems to abate across the life course is encouraging.

## Supplemental Material

SJP902914_Supplemental_material – Supplemental material for Poor family relationships in adolescence as a risk factor of in-patient psychiatric care across the life course: A prospective cohort study Click here for additional data file. Supplemental material, SJP902914_Supplemental_material for Poor family relationships in adolescence as a risk factor of in-patient psychiatric care across the life course: A prospective cohort study by Susanne Alm, Sara Brolin Låftman, Fredrik Sivertsson and Hannes Bohman in Scandinavian Journal of Public Health
