# Supplementary material for: Poor family relationships in adolescence as a risk factor of in-patient psychiatric care across the life course: A prospective cohort study
Source: Scand J Public Health. 2020 Feb 3;48(7):726–32. doi: 10.1177/1403494820902914 (PMC7605045; doi:10.1177/1403494820902914)
Supplement: SJP902914_Supplemental_material – Supplemental material for Poor family relationships in adolescence as a risk factor of in-patient psychiatric care across the life course: A prospective cohort study [file SJP902914_Supplemental_material.pdf]

Table AI. Sensitivity analysis. Associations between family relations in adolescence and in-patient psychiatric care at ages 16–25, 26–35, 36–45 and 46–55 years. Odds ratios and 95% confidence intervals (95% CI) from binary logistic regressions. Results statistically significant at the 5%-level are reported in bold. n=2500 (all models).

	Adjusted	
	OR	95% CI
**16-25 years**		
Family relations		
Good (ref.)	1.00	-
Intermediate	**2.36**	1.21-4.59
Poor	**5.06**	2.27-11.30
**26-35 years**		
Family relations		
Good (ref.)	1.00	-
Intermediate	1.74	0.94-3.22
Poor	**3.49**	1.60-7.60
**36-45 years**		
Family relations		
Good (ref.)	1.00	-
Intermediate	1.57	0.97-2.55
Poor	**2.02**	1.01-4.06
**46-55 years**		
Family relations		
Good (ref.)	1.00	-
Intermediate	1.40	0.88-2.22
Poor	1.70	0.84-3.47

Table AII. Sensitivity analysis. Associations between family relations in adolescence and in-patient psychiatric care in young adulthood (16-25 years), breaking down the index of family relations into its four components: Mother-child relation, father-child relation, sibling relations and mother-father relation. Gender, SES, household economic poverty, contact with child services, parental drug abuse and parental mental illness controlled for in models reporting adjusted effects. Odds ratios (OR) and 95% confidence intervals (95% CI) from binary logistic regressions. Results statistically significant at the 5%-level are reported in bold. (n=2614)

		Unadjusted		Adjusted	
	% with diagnosis (n)	OR	95% CI	OR	95% CI
Family relations					
Mother-child					
Good (ref.)	1.9 (31)	1.00	-	1.00	-
Intermediate	4.2 (40)	**2.22**	1.38-3.58	**2.12**	1.31-3.42
Poor	7.0 (3)	**3.83**	1.12-13.04	2.97	0.84-10.58
Father-child					
Good (ref.)	1.5 (22)	1.00	-	1.00	-
Intermediate	4.1 (42)	**2.89**	1.71-4.86	**2.75**	1.62-4.65
Poor	10.1 (10)	**7.53**	3.46-16.39	**5.82**	2.57-13.19
Sibling relations					
Good (ref.)	1.9 (19)	1.00	-	1.00	-
Intermediate	3.3 (42)	1.69	0.98-2.93	1.59	0.91-2.76
Poor	3.7 (13)	1.95	0.95-4.00	1.73	0.84-3.58
Mother-father					
Good (ref.)	1.9 (32)	1.00	-	1.00	-
Intermediate	4.2 (35)	**2.27**	1.39-3.69	**2.02**	1.25-3.32
Poor	7.6 (7)	**4.26**	1.83-9.93	**3.43**	1.44-8.14

Table AIII. Sensitivity analysis. Negative binomial regression of in-patient psychiatric care in age 16-55 years. Incidence rate ratios (IRR) and 95% CIs. Results statistically significant at the 5%-level are reported in bold. n=2500

	Unadjusted		Adjusted	
	IRR	95% CI	IRR	95% CI
Family relations				
Good (ref.)	1.00	-	1.00	-
Intermediate	**2.12**	1.28-3.50	**2.58**	1.56-4.26
Poor	**4.78**	2.02-11.36	**3.30**	1.32-8.26
Gender				
Males (ref.)	1.00	-	1.00	-
Females	0.62	0.38-1.02	**0.62**	0.38-0.99
Household social class				
Upper non-manual (ref.)	1.00	-	1.00	-
Interm./lower non manual/entrepreneur/ farmer	**2.01**	1.05-3.82	1.60	0.86-3.00
Manual worker	**3.27**	1.70-6.28	1.44	0.73-2.86
Household economic poverty	**3.91**	1.89-8.11	**3.04**	1.30-7.10
Contact with child services	4.59	0.70-29.97	0.91	0.08-10.16
Parental alcohol abuse	5.55	0.71-43.24	1.27	0.09-17.80
Parental mental illness	**4.34**	1.01-18.72	2.22	0.50-9.84

Table AIV. Sensitivity analysis. Associations between family relations in adolescence and in-patient psychiatric care in adulthood (16-55 years), breaking down the dependent variable into four different types of psychiatric disorders. Gender, SES, household economic poverty, contact with child services, parental drug abuse and parental mental illness controlled for in models reporting adjusted effects. Odds ratios (OR) and 95% confidence intervals (95% CI) from binary logistic regressions. Results statistically significant at the 5%-level are reported in bold. (n=2500)

		Unadjusted		Adjusted	
	% with diagnosis (n)	OR	95% CI	OR	95% CI
**Drug-related disorder (n=95)**					
Family relations:					
Good (ref.)	2.7 (33)	1.00		1.00	
Intermediate	4.4 (47)	**1.65**	1.05-2.60	1.58	1.00-2.50
Poor	6.9 (15)	**2.65**	1.41-4.97	**2.17**	1.12-4.20
**Mood/anxiety disorder (n=78)**					
Family relations:					
Good (ref.)	2.5 (30)	1.00		1.00	
Intermediate	3.7 (39)	1.50	0.93-2.43	1.48	0.91-2.41
Poor	4.1 (9)	1.70	0.80-3.64	1.54	0.71-3.37
**Psychosis and related disorders (n=29)**					
Family relations:					
Good (ref.)	0.6 (7)	1.00		1.00	
Intermediate	1.5 (16)	**2.63**	1.08-6.42	**2.52**	1.03-6.18
Poor	2.8 (6)	**4.89**	1.63-14.69	**4.50**	1.48-13.74
**Other (n=47)**					
Family relations:					
Good (ref.)	1.3 (16)	1.00		1.00	
Intermediate	2.3 (25)	1.80	0.96-3.39	1.72	0.91-3.25
Poor	2.8 (6)	2.12	0.82-5.49	1.82	0.69-4.83
